# The role of homocysteine levels as a risk factor of ischemic stroke events: a systematic review and meta-analysis

**DOI:** 10.3389/fneur.2023.1144584

**Published:** 2023-05-12

**Authors:** Rizaldy Taslim Pinzon, Vincent Ongko Wijaya, Vanessa Veronica

**Affiliations:** ^1^Faculty of Medicine, Duta Wacana Christian University, Yogyakarta, Indonesia; ^2^Neurology Department, Bethesda Hospital, Yogyakarta, Indonesia

**Keywords:** homocysteine, ischemic stroke, risk factor, systematic review, meta-analysis

## Abstract

**Introduction:**

Among numerous risk factors, homocysteine (Hcy) has been linked to cerebral infarction; however, results have been inconsistent. This review aimed to conduct a meta-analysis of published studies to investigate the relationship between plasma Hcy levels and the risk of ischemic stroke.

**Methods:**

A systematic literature search was conducted until November 2022 to obtain articles reporting Hcy levels in ischemic stroke patients. Review Manager software was used to perform all statistical analyses (version 5.3).

**Results:**

Initial investigation yielded 283 articles. The final evaluation included 21 articles, including two prospective studies, one retrospective cohort, and 18 case–control studies. These studies included 9888 participants, of which 5031 were admitted patients with ischemic stroke. An integrated analysis revealed that ischemic stroke patients had significantly higher levels of Hcy than controls (mean difference (MD) = +3.70, 95% confidence interval (CI) = 2.42–5.81, *p* < 0.001).

**Conclusion:**

This meta-analysis and systematic review indicate that ischemic stroke patients have significantly higher homocysteine levels than controls. Detecting hyperhomocysteinemia and reducing homocysteine levels should be explored among individuals at increased risk for ischemic stroke.

## Introduction

The cerebrovascular disease has emerged as the leading cause of disability and the second leading cause of death worldwide. Ischemic stroke is one of the most common cerebrovascular diseases, constituting 85% of all strokes (1). Older age, gender, hypertension, diabetes mellitus, hypercholesterolemia, and smoking are the traditional risk factors for cerebrovascular disease (2). Among a variety of risk factors, studies have found that homocysteine (Hcy) is an independent risk factor and correlated with cerebral infarction due to intracranial small-vessel disease and extracranial vascular disease, including myocardial infarction and peripheral artery disease (3–6).

Homocysteine (Hcy) is a naturally sulfhydryl-containing amino acid and is closely linked with endothelial dysfunction and extracellular matrix proliferation that may cause vessel damage (7). Recent studies reported a possible association between hyperhomocysteinemia and thrombotic vascular events, including ischemic stroke (8–10), but these studies have suggested mixed conclusions, and the mechanism by which homocysteine affects stroke prognosis is still unclear. In recent years, researchers have conducted numerous case–control studies to explore the possible correlation between Hcy and cerebral infarction (11, 12). Nevertheless, the results have been inconsistent. Most of the published studies on Hcy and ischemic stroke only had modest sample sizes and were not well-designed, affecting their significance. Current guidelines did not recommend any treatment for Hcy levels. However, if the role of Hcy levels may affect stroke outcomes, controlling Hcy levels may be a novel treatment option for stroke treatment and prevention.

Therefore, the aim of this review was to perform a meta-analysis of published studies to assess the relationship between plasma Hcy levels and the risk of ischemic stroke.

## Methods

This review was conducted according to the Preferred Reporting Items for Systematic Review and Meta-Analysis (PRISMA) guidelines (13).

### Literature search and selection criteria

Initially, three independent reviewers screened the databases of the included studies on PubMed, and MedRvix up to November 2022, using specific keywords: “ischemic stroke” OR “cerebral infarct” AND “homocysteine.” We used the following criteria to identify eligible studies that investigated the association between Hcy levels and ischemic stroke: (1) studies that reported the relationship between baseline plasma Hcy levels (measured at admission) and patients with ischemic stroke and (2) studies that compared ischemic stroke patients and healthy controls (case–control). The literature search was also restricted to English-language articles only. The exclusion criteria were as follows: (1) single-arm trials (no control/comparison group); (2) outcomes out of interest (studies that did not estimate the mean differences between ischemic stroke patients and healthy controls); and (3) data cannot be extracted (incomplete data). The primary outcome was the differences in the plasma Hcy levels between ischemic stroke patients and the control group, and the secondary outcome was the differences in the plasma Hcy levels between male and female ischemic stroke patients.

### Data extraction and quality assessment

In total, three authors independently screened and examined the titles and abstract, followed by a full-text review using the inclusion and exclusion criteria. In the event of disagreement between the three authors, the main author would help to resolve the issue and make a final decision. Studies that entirely fulfilled our inclusion criteria were retrieved and additional articles were added based on the bibliography of the articles retrieved through the outlined search strategy. If the reviewers could not reach an agreement, the first author will be consulted for the final decision.

We extracted and tabulated the following data: author(s), year of publication, study design, country of origin, baseline characteristics, homocysteine levels (mean ± standard deviation), and clinical outcomes. The quality of each included study was assessed using the Oxford Center for Evidence-Based Medicine Quality ratings and classified the evidence ratings ranged from one to five, with one representing high-quality studies such as randomized controlled trials (RCT) and five representing case reports (14).

### Statistical analysis

All the analyses were performed using Review Manager software (version 5.3). Standardized mean difference (SMD) with a 95% confidence interval (CI) was used for continuous variables to compare the homocysteine levels between groups. The I2 tests measured heterogeneity among studies, and studies with I2 higher than 50% were considered to have high heterogeneity. A fixed-effects model was used when there was no significant heterogeneity among studies; otherwise, a random-effects model was used when data were considered heterogeneous. Two-sided *P*-values of < 0.05 were regarded as statistical significance (15, 16).

## Results

### Study characteristics

**Figure 1:**
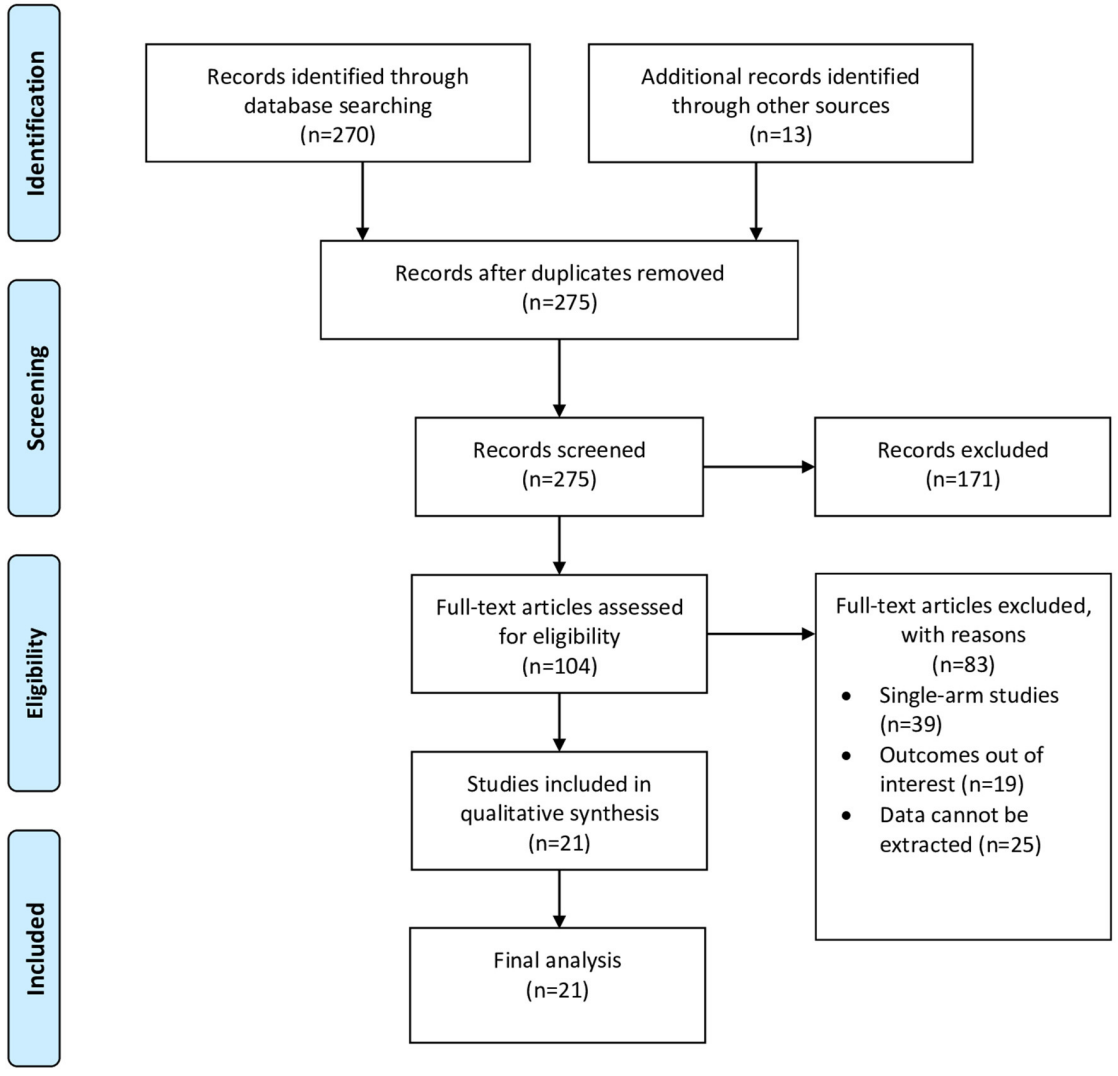
PRISMA flow chart.

The search strategy initially generated 283 articles. After removing duplicates and abstract screening, 104 full-text articles were assessed for eligibility. Finally, 21 articles were included in the final review, including two prospective studies, one retrospective cohort, and 18 case–control studies. Figure 1 shows the PRISMA flow chart of study selection.

This process resulted in the selection of 21 studies involving a total of 9888 participants, of whom 5031 were patients admitted with ischemic stroke, for the meta-analysis. Of the included studies, first author, publication year, total sample participants, country location, ethnicity, age, and study quality level were assessed. The studies included in the meta-analysis were generally of moderate quality rating (Table 1).

**Table 1:** Baseline characteristics of patients in the included studies.

**Authors**	**Study type**	**Country location**	**No. of participants, (n)**	**Ethnicity**	**Age, Median (IQR, y) or Mean ±SD**	**Study quality level**
Alfieri et al. (17)	Prospective cohort	Brazil	352	Caucasians	IS group: 67.7 **±** 12.1, Control group: 63.1 **±** 11.3	2
Jin et al. (18)	Case-control	China	3575	Asians	IS group: 62.71 ± 11.86, Control group: 50.82 ± 8.87	3
Ma et al. (19)	Retrospective Cohort	China	314	Asians	IS group: 53.8 ± 6.2, Control group: 54.0 ± 7.0	3
Shademan et al. (20)	Case-control	Turkey	240	Asians	IS group: 58.2 ± 8.5, Control group: 55.1 ± 6.6	3
Yurekli et al. (21)	Prospective trial	Turkey	118	Asians	IS group: 61.07 ± 6.28, Control group: 58.71 ± 5.66	2
Wang et al. (22)	Case-control	China	202	Asians	IS group: 61.07 ± 11.56, Control group: 62.49 ± 8.93	3
Kawamoto et al. (23)	Case-control	Japan	91	Asians	IS group: 81 ± 7, Control group: 79 ± 6.5	3
Yoldas et al. (24)	Case-control	Turkey	80	Asians	IS group: 69 ± 11, Control group: 70 ± 9	3
Salem-Berrabah et al. (25)	Case-control	Tunisia	147	Africans	IS group: 57.62, Control group: 30 to 70 years	3
Omrani et al. (26)	Case-control	Iran	186	Arabs	IS group: 62.2 ± 9.8, Control group: 61.8 ± 9.9	3
Wei et al. (27)	Case-Control	China	1108	Asians	IS group: 59.34 ± 9.25, Control group: 59.88 ± 10.12	3
Luo et al. (28)	Case-Control	China	601	Asians	IS group: 60.70 ± 12.33, Control group: 60.17 ± 10.32	3
Modi et al. (29)	Case-Control	India	87	Asians	NR	3
Xiao et al. (30)	Case-Control	China	304	Asians	IS group: 60.37 ± 12.02, Control group: 60.45 ± 12.23	3
Narayan et al. (31)	Case-Control	India	175	Asians	IS group: 53.3 ± 14.6, Venous stroke group: 30.9 ± 6.6, Control group: 51.8 ± 9.3	3
Al-Allawi and Jubrael. (32)	Case-Control	Iraq	120	Arabs	IS group: 60, Control group: 62	3
Lu et al. (33)	Case-Control	China	320	Asians	IS group: 63.91 ± 11.49, Control group: 61.65 ± 11.47	3
Zheng et al. (34)	Case-Control	China	418	Asians	MCA stroke group: 64 ± 12, CA stroke group: 62 ± 11, BA stroke group: 60 ± 13, Control group: 64 ± 11	3
Chen et al. (35)	Case-Control	China	610	Asians	IS group: 64.40 ± 12.90, Control group: 65.16 ± 11.95	3
Zhou and Qi. (36)	Case-Control	China	216	Asians	IS group: 66.32 ± 11.51, Control group: 64.46 ± 12.77	3
Chen et al. (37)	Case-Control	China	730	Asians	IS group: 65.7 ± 11.5, Control group: 66.3 ± 10.2	3

BA, Basilar Artery; IS, Ischemic Stroke; MCA, Middle Cerebral Artery; NR, Not Reported in detail; PCA, Posterior Cerebral Artery.

### Homocysteine levels in patients with ischemic stroke

**Figure 2:**
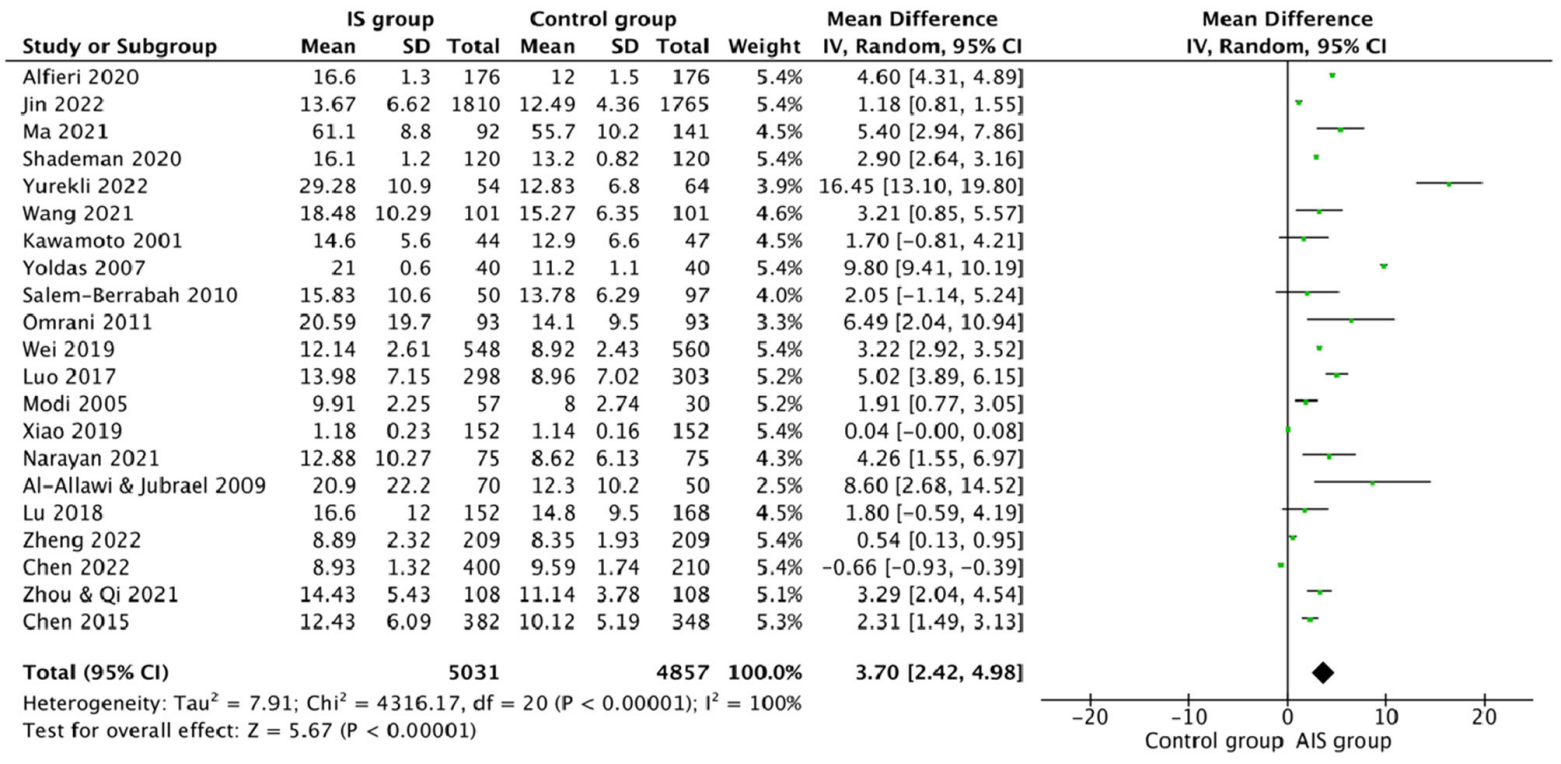
Forest plots for comparing plasma Hcy levels between ischemic stroke (AIS) patients and controls.

**Figure 3:**
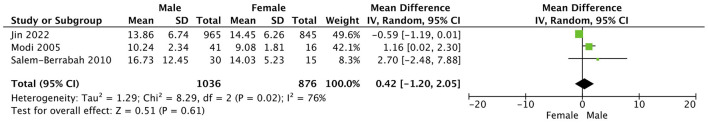
Forest plots of sex differences comparing plasma Hcy levels in ischemic stroke (AIS) patients.

This study compared the differences in the plasma Hcy levels between ischemic stroke patients and the control group, and other features were listed (Table 2). There was high heterogeneity among the studies reporting differences in Hcy levels between ischemic stroke patients and control (*I*^2^ = 100%). Thus, a random-effects model was used to analyze the data. An incorporated analysis showed that the AIS patients had significantly higher levels of Hcy compared to the controls (MD = +3.70, 95% CI = 2.42–5.98, *p* < 0.001) (Figure 2). Additional analysis of sex differences showed that male acute ischemic stroke patients had higher levels of Hcy compared to female patients (MD = +0.42, 95% CI = −1.20–2.05, *p* = 0.61) (Figure 3).

**Table 2:** Patients group and clinical characteristics of patients in the included studies.

**Authors**	**Ischemic stroke group (no. of patients)**	**Control group (no. of patients)**	**Ischemic stroke group Hcy levels, μmol/L (Mean ±SD)**	**Control group Hcy levels, μmol/L (Mean ±SD)**	**Stroke subtypes**	**Follow-up**	**Covariates adjustment**	**Other outcomes**
Alfieri et al. (17)	176	176	16.6 ± 1.3	12.0 ± 1.5	NR	3 months	Age, sex, ethnicity, BMI, smoking, and previous medications (antihypertensive, hypolipemiant, and hypoglycemic drugs)	The main findings of the study are that IS associated with increased WBC counts, high hsCRP, IL-6, lipid hydroperoxides (LOOH), NOx, homocysteine, ferritin, ESR, glucose, and insulin, and lowered iron, 25(OH)D level, total cholesterol, and HDL cholesterol
Jin et al. (18)	1810 (male: 965; female: 845)	1765 (male: 570; female: 1195)	13.67 ± 6.62 (male: 13.86 ± 6.74; female:14.45 ± 6.26)	12.49 ± 4.36 (male: 11.93 ± 5.46; female: 12.86 ± 5.74)	large-artery atherosclerosis (LAA)	NR	Age, sex	In LAA-IS patients, the TT homozygous genotype correlated with an increased risk of developing LAAIS, The plasma homocysteine level was genotype-dependent according to the following trend: TT > CT > CC
Ma et al. (19)	92 hypertensive patients with IS	114 hypertensive patients without IS	61.1 ± 8.8	55.7 ± 10.2	NR	6 months	NR	In hypertensive patients with IS, serum cytotoxic T lymphocyte-associated antigen-4 (CTLA-4), ischemia-modified albumin (IMA), lipoprotein-associated phospholipase A2 (Lp-PLA2), glial fibrillary acidic protein (GFAP), and homocysteine (HCY) levels were significantly higher compared to controls (*p* < 0.05)
Shademan et al. (20)	120	120	16.1 ± 1.20	13.2 ± 0.82	NR	NR	Blood pressure, glucose, and cholesterol	The mean serum levels of apolipoprotein B 48, interleukin-1β, and Homocysteine, were significantly increased in the experimental group compared to the control group with a *p*-value of 0.001
Yurekli et al. (21)	54	64	29.28 ± 10.9	12.83 ± 6.8	NR	24 h after admission	NR	Compared to the control group, IS patients had lower serum vitamin D (*p* < 0.0001) and brain-derived neurotrophic factor (*p* < 0.0001) levels and higher homocysteine levels (*p* < 0.0001). There was a correlation between vitamin D levels and BDNF levels in patients with IS
Wang et al. (22)	101	101	18.48 ± 10.29	15.27 ± 6.35	NR	NR	Age, sex, BMI, TG, TC, HDL, and LDL	Serine hydroxymethyl transferase 1 (SHMT1) gene hypermethylation was significantly associated with high Hcy concentration in ischemic stroke patients
Kawamoto et al. (23)	44	47	14.6 ± 5.6	12.9 ± 6.6	NR	NR	Age, gender, albumin, creatinine, hypertension, diabetes, smoking, BMI, TG, TC, HDL, and uric acid	There was an association between elevated Hcy levels (>10 μ**mol/L)** and IS among the elderly Japanese
Yoldas et al. (24)	40	40	21.0 ± 0.6	11.2 ± 1.1	NR	NR	NR	Subjects with stroke have higher circulating serum hsCRP and homocysteine levels
Salem-Berrabah et al. (25)	50 (male: 30; female: 15)	97 (male: 50; female: 46)	15.83 ± 10.60 (male: 16.73 ± 12.45; female:14.03 ± 5.23)	13.78 ± 6.29 (male: 14.7 ± 6.03; female: 12.78 ± 6.47)	NR	NR	NR	In Tunisian subjects, the risk of developing ischemic stroke in hyperhomocysteinemic subjects was 2.4 times more than in subjects with normal Hcy levels (OR = 2.4; 95% CI: 1.13–5.06; *p* < 0.05).
Omrani et al. (26)	93	93	20.59 ± 19.7	14.1 ± 9.5	NR	NR	Smoking	In this study, 41% of patients had hyperhomocysteinemia. Hcy plasma levels in the acute phase of ischemic stroke (within 24 h) were significantly higher than normal limits
Wei et al. (27)	548	560	12.14 ± 2.61	8.92 ± 2.43	NR	NR	Gender, age, smoker, diabetes and hypertension	Homocysteine was significantly higher in ischemic stroke patients than in the controls (*p* < 0.001). Higher levels of homocysteine were reported in patients with ischemic stroke who had the rs2666433AA genotype compared to those who carried the rs2666433 GG+GA genotypes (*p* < 0.001)
Luo et al. (28)	298	303	13.98 ± 7.15	8.96 ± 7.02	NR	NR	Gender, age, smoking situation, diabetes, hypertension	Homocysteine was significantly higher in ischemic stroke patients than in the controls (*p* < 0.001)
Modi et al. (29)	57 (male: 41; female: 16)	30 (male: 22; female: 8)	9.91 ± 2.25 (male: 10.24 ± 2.34; female: 9.08 ± 1.81)	8.00 ± 2.74 (male: 8.45 ± 2.72; female: 6.79± 2.60)	NR	NR	Gender, smoking, hypertension, obesity	Hyperhomocysteinemia is a significant independent risk factor for ischemic stroke (*p* < 0.01). A considerable positive correlation was also found between hypertension, smoking, and elevated levels of homocysteine
Xiao et al. (30)	152	152	1.18 ± 0.23	1.14 ± 0.16	NR	NR	Telomere length, glucose, TC, HDL	Homocysteine was significantly higher in ischemic stroke patients than in the controls (*p*: 0.047). Telomere length and homocysteine (HCY) were inversely associated in ischemic stroke patients (*r* = −0.176, *p*: 0.03)
Narayan et al. (31)	75 IS patients and 25 venous stroke patients	75	IS group: 12.88 ± 10.27, venous stroke group: 8.08 ± 4.17	8.62 ± 6.13	Ischemic stroke and venous stroke	NR	NR	Homocysteine was significantly higher in ischemic stroke patients than in the controls (*p*: 0.02). Ischemic stroke and venous stroke patients were younger than 45 years old
Al-Allawi and Jubrael. (32)	70	50	20.9 ± 22.2	12.3 ± 10.2	NR	NR	NR	Homocysteine was significantly higher in ischemic stroke patients than in the controls (*p*: 0.02). TT and CT genotypes had greater homocysteine levels than the CC genotype (*p* < 0.001 and *p*: 0.04, consecutively). No interquartile ranges for age were available
Lu et al. (33)	152	168	16.628 ± 12.0426	14.78 ± 9.494	NR	NR	Age, gender, smoking, alcohol consumption, SBP, DBP, blood glucose, TC, TG, LDL, HDL, UA, plasma fibrinogen level	NR
Zheng et al. (34)	209	209	MCA stroke group: 8.89 ± 2.32, PCA stroke group: 7.99 ± 2.20, BA stroke group: 8.09 ± 2.54	8.35 ± 1.93	MCA, PCA, and BA stroke	NR	NR	MCA stroke patients had significantly higher homocysteine levels than PCA (*p* = 0.016) and BA stroke patients (*p*: 0.013)
Chen et al. (35)	400	210	8.93 ± 1.32	9.59 ± 1.74	NR	NR	NR	NR
Zhou and Qi. (36)	108	108	14.43 ± 5.43	11.14 ± 3.78	NR	NR	NR	Homocysteine was significantly higher in ischemic stroke patients than in the controls (*p* < 0.001)
Chen et al. (37)	382	348	12.43 ± 6.09	10.12 ± 5.19	NR	NR	NR	Homocysteine was significantly higher in ischemic stroke patients than in the controls (*p* < 0.001). Homocysteine levels were statistically lower in ischemic stroke patients with the GG or AG genotype than in those with the AG or AA genotype

BA, Basilar Artery; IS, Ischemic Stroke; MCA, Middle Cerebral Artery; NR, Not Reported in detail; PCA, Posterior Cerebral Artery; SBP, Systolic Blood Pressure; DBP, Diastolic Blood Pressure, TC, Total Cholesterol, TG, Triglycerides, LDL, low-density lipoprotein, HDL, high-density lipoprotein, UA, uric acid.

## Discussion

Homocysteine is a non-dietary amino acid that can be transformed into cysteine or recycled into methionine, a necessary amino acid, with the assistance of certain B vitamins. Normal homocysteine ranges in men and women vary between 5 and 10 micromol/L (micromoles per liter). If homocysteine levels surpass 10 micromol/L, this condition is called hyperhomocysteinemia (38, 39). Data from our systematic review and meta-analysis suggested the following: (1) patients with ischemic stroke had greater homocysteine levels than controls and (2) homocysteine could be an independent risk factor for the outcome of ischemic stroke patients. Homocysteine levels are often classified as mild (slightly above 10 micromol/L), moderate (16–30 micromol/L), intermediate (31–100 micromol/L), and severe (above 100 micromol/L) (40). Even mild hyperhomocysteinemia may increase the risk for ischemic stroke, as demonstrated by numerous studies in this systematic review and meta-analysis (18, 23, 25, 27, 28, 31, 36, 37). In total, three studies did not find homocysteine levels that meet the criteria for hyperhomocysteinemia, but all showed a tendency for greater homocysteine levels in stroke patients compared to controls (29, 30, 34). A prior study concludes that the effect of blood homocysteine level on stroke severity and outcome begins to appear between 8 and 10 micromol/L (41).

A higher homocysteine level raises the risk of vascular diseases, including stroke. Conversely, a decrease in homocysteine levels is correlated with a reduced risk of ischemic stroke (42). Elevated homocysteine levels can lead to stroke through a variety of pathways. Homocysteine promotes the transcription of the factor in the neural tissue, which enhances inflammation by elevating the concentration of inflammatory cytokines. Homocysteine accumulation within cells has been demonstrated to impede methyltransferases, reduce deoxyribonucleic acid (DNA) repair, and promote apoptosis. Autooxidation of homocysteine metabolites generates H_2_O_2_ and results in necrotic cell death (43, 44). Plasma homocysteine levels are frequently associated with the development of atherosclerosis and the degradation of vascular endothelium. Homocysteine induces the formation of serine elastase in vascular smooth muscle cells, which results in elastolysis by dissolving the extracellular matrix and generating reactive oxygen species (45).

One of the studies in this systematic review and meta-analysis comparing large-artery atherosclerosis stroke patients and healthy controls found a significant difference in homocysteine blood levels (18). Similar results were reported in a previous meta-analysis comparing homocysteine blood levels among acute stroke patients (2,243 patients) and a control group (871 patients). Hyperhomocysteinemia is most often related to the subtypes “small-vessel occlusion” and “large-artery atherosclerosis” (46).

Depending on their locations, individuals with middle cerebral artery (MCA) stroke had significantly higher homocysteine levels than patients with the posterior cerebral artery (PCA) and basilar artery (BA) stroke (34). Higher homocysteine levels in MCA stroke patients compared to BA stroke patients may be indicative of a higher risk of post-stroke cardiovascular disorders in MCA stroke patients related to a hypercoagulable state (47).

Hyperhomocysteinemia is also a risk factor for other stroke subtypes, including intracerebral hemorrhage, the second-leading subtype of stroke (48). In an earlier meta-analysis involving 667 patients with intracerebral hemorrhage, 1821 patients with ischemic stroke, and 2500 healthy controls, homocysteine levels in intracerebral hemorrhage patients were significantly higher than in healthy controls, indicating that the exact pathophysiology of intracerebral hemorrhage inevitably involves increased homocysteine levels (49). The plasma homocysteine level was found to be an exacerbating factor in atherosclerosis, resulting in the pathogenesis of endothelial degeneration and vessel wall necrosis, which could increase the risk of ischemic stroke as well as intracerebral hemorrhage (50). Additionally, a raised homocysteine level was significantly associated with an increased risk of recurrent stroke within 15 months after the initial cerebrovascular event (51). A plasma homocysteine level above the 75th percentile 3 months following an ischemic stroke was predictive of vascular events, including stroke recurrence (52).

Vitamin B deficiency is a potential challenge that might impair homocysteine metabolism and lead to hyperhomocysteinemia (53). Nonetheless, vitamin B supplementation and homocysteine reduction remain the subjects of several debates. In the Vitamins to Prevent Stroke (VITATOPS) trial, daily B vitamins supplementation did not appear to be over the placebo in reducing the incidence of major vascular events (54). It was hypothesized that antiplatelet therapy, administered to approximately 80% of patients in the VITATOPS trial, might have modulated the beneficial impact of B vitamins on homocysteine levels. Patients who were receiving antiplatelet therapy at the baseline were separated from those who were not in the *post*-*hoc* analysis. There was no significant difference in the primary outcome between the placebo and vitamin B groups in patients receiving antiplatelet medication at the baseline (14.8% vs. 15.7%). However, for patients who did not receive antiplatelet therapy at the baseline, vitamin B treatment correlated with a significant reduction in primary outcome events (16.8% vs. 21.0%) (55). According to the Vitamin Intervention for Stroke Prevention (VISP) trial, moderate homocysteine reduction did not affect vascular outcomes (56). However, there were a few issues with the VISP trial. It appears that VISP gave too much cobalamin in the low-dose vitamin arm of the study (6 mcg daily; at least the recommended daily intake [RDI] or, by some measures, three times the RDI) as well as insufficient cobalamin in the high-dose vitamin arm (400 mcg daily) for geriatric patients (57). A dose–response study revealed that geriatric patients with cobalamin levels below 221 pmol/L require 1000 mcg daily for optimal absorption (58). It became clear that the ability to absorb sufficient levels of cobalamin was the primary determinant of response to vitamin therapy in homocysteine reduction. Mecobalamin, one of the active analogs of cobalamin, has been shown to reduce plasma homocysteine concentrations. An earlier study revealed that after 4 weeks, 8 weeks, 3 months, and 6 months of supplementation, the homocysteine level in the group receiving 500 μg of mecobalamin three times a day was lower than in the group receiving only conventional therapy. In addition, the treatment group had significantly higher scores on the National Institutes of Health Stroke Scale (NIHSS) after 3 and 6 months of mecobalamin supplementation than the control group. (59). Similar to cobalamin, folate is an essential regulator in the homocysteine metabolic process; a previous meta-analysis comprising 14 randomized controlled trials with a total of 39,420 participants showed that homocysteine reduction after folic acid supplementation was significantly higher in regions without folate fortification than in regions with folate fortification (60).

Despite all the contrasts, multiple studies indicate that daily vitamin B intake has a strong preventive effect against stroke or transient ischemic attack (61). Reducing homocysteine levels prior to the onset of atherosclerosis may have preventative benefits for vascular events. In other words, homocysteine must be decreased as promptly as possible. Yet another issue that must be addressed is attempting to determine the impact of modifiable risk factors, including hyperhomocysteinemia, on medical care, such as suggesting homocysteine-lowering interventions, including supplementation with vitamin B, to decrease the probability of stroke or achieving better prognosis of stroke patients.

There were some limitations in our study. (1) Most of the included studies only measured homocysteine levels at hospital admission. There was a lack of data on changes in homocysteine levels during follow-up. Therefore, further studies assessing the average time of measurement of homocysteine levels following an ischemic stroke or during hospitalization would help understand whether homocysteine is a risk factor or a consequence of stroke. (2) Our primary outcome was to compare the homocysteine levels between the ischemic stroke and control group. Further studies are needed to analyze other covariates (different types of strokes and comorbidity) or predict the risk estimates of hyperhomocysteinemia.

## Conclusion

This meta-analysis and systematic review indicate that ischemic stroke patients have significantly higher homocysteine levels than controls. Detecting hyperhomocysteinemia and reducing homocysteine levels should be explored among individuals at increased risk for ischemic stroke.

## Data availability statement

The original contributions presented in the study are included in the article/supplementary material, further inquiries can be directed to the corresponding author.

## Author contributions

RP: supervision, study concept, writing of the initial draft, and data extraction. VW: writing of the initial draft, data extraction, analysis, and interpretation. VV: full-text review, manuscript preparation, and data extraction and analysis. All authors contributed to the article and approved the submitted version.

## References

[1] Bamford J Sandercock P Dennis M . A prospective study of acute cerebrovascular disease in the community: the oxfordshire community stroke project 1981–86. (1 Methodology, demography and incident cases of first-ever stroke. J Neurol Neurosurg Psychiatry. (1988) 51:1373–80. 10.1136/jnnp.51.11.1373 3266234 PMC1032805

[2] Go AS Mozaffarian D Roger VL . Heart disease and stroke statistics−2013 update: a report from the American Heart Association. Circulation. (2013) 127:e6–245. 23239837 10.1161/CIR.0b013e31828124ad PMC5408511

[3] Jeon SB Kang DW Kim JS Kwon SU. Homocysteine, small- vessel disease, and atherosclerosis: an MRI study of 825 stroke patients. Neurology. (2014) 83:695–701. 10.1212/WNL.0000000000000720 25031284

[4] Piao X Wu G Yang P Shen J De A Wu J . Association between homocysteine and cerebral small vessel disease: a meta-analysis. J Stroke Cerebrovasc Dis. (2018) 27:2423–30. 10.1016/j.jstrokecerebrovasdis.2018.04.035 29801814

[5] Nygård O Nordrehaug JE Refsum H Ueland PM Farstad M Vollset SE. Plasma homocysteine levels and mortality in patients with coronary artery disease. N Engl J Med. (1997) 337:230–6. 10.1056/NEJM199707243370403 9227928

[6] Clarke R Daly L Robinson K. Hyperhomocysteinemia: an independent risk factor for vascular disease. N Engl J Med. (1991) 324:1149–55. 10.1056/NEJM199104253241701 2011158

[7] Spence JD. Homocysteine-lowering therapy: a role in stroke prevention? Lancet Neurol. (2007) 6:830–8. 10.1016/S1474-4422(07)70219-3 17706567

[8] Miwa K Tanaka M Okazaki S Yagita Y Sakaguchi M Mochizuki H . Increased total homocysteine levels predict the risk of incident dementia independent of cerebral small-vessel diseases and vascular risk factors. J Alzheimers Dis. (2016) 49:503–13. 10.3233/JAD-150458 26484913

[9] Shi Z Guan Y Huo YR Liu S Zhang M Lu H . Elevated total homocysteine levels in acute ischemic stroke are associated with long-term mortality. Stroke. (2015) 46:2419–25. 10.1161/STROKEAHA.115.009136 26199315 PMC4542568

[10] Han L Wu Q Wang C. Homocysteine, ischemic stroke, and coronary heart disease in hypertensive patients: a population-based, prospective cohort study. Stroke. (2015) 46:1777–86. 10.1161/STROKEAHA.115.009111 26038522

[11] Yin SW Ding SW Dai JY. The significance of serum homocysteine levels in 65 patients with cerebral infarction. Chin J Geriatr. (2004) 23:203.

[12] Li N Zhang YG Guo XH . Study on the association between homocysteine and the size of cerebral infarction. Chin J Rehabil Theory Pract. (2005) 11:370–1.

[13] Shamseer L Moher D Clarke M. Preferred reporting items for systematic review and meta-analysis protocols (PRISMA-P) 2015: elaboration and explanation. BMJ. (2015) 350:g7647. 10.1136/bmj.g7647 25555855

[14] OCEBM Levels of Evidence Working Group. The Oxford Levels of Evidence 2. Oxford: Oxford Centre for Evidence-Based Medicine. (2011).

[15] Higgins JPT Green S editors,. 7.7.3.5. Medians and interquartile ranges. In: *Co- chrane Handbook for Systematic Reviews of Interventions*. Version 5.1.0. Oxford (UK): Cochrane Collaboration) 2011. Available online at: https://handbook-5-1.cochrane.org/chapter_7/7_7_3_5_mediansand_interquartile_ranges.htm (accessed November 10, 2022).

[16] Higgins JPT Altman DG Gotzsche PC. The Cochrane Collaboration's tool for assessing risk of bias in randomized trials. BMJ. (2011) 343:d5928. 10.1136/bmj.d5928 22008217 PMC3196245

[17] Alfieri DF Lehmann MF Flauzino T de Araújo MCM Pivoto N Tirolla RM . Immune-inflammatory, metabolic, oxidative, and nitrosative stress biomarkers predict acute ischemic stroke and short-term outcome. Neurotox Res. (2020) 38:330–43. 10.1007/s12640-020-00221-0 32415527

[18] Jin M Wang N Li X Zhang H Zhou J Cong M . Relationship between MTHFR C677T, homocysteine, and ischemic stroke in a large sample of the Han Chinese population. Medicine. (2022) 101:e30562. 10.1097/MD.0000000000030562 36197177 PMC9509028

[19] Ma J Shen L Bao L Yuan H Wang Y Liu H . novel prognosis prediction model, including cytotoxic T lymphocyte-associated antigen-4, ischemia-modified albumin, lipoprotein-associated phospholipase A2, glial fibrillary acidic protein, and homocysteine, for ischemic stroke in the Chinese hypertensive population. J Clin Lab Anal. (2021) 35:e23756. 10.1002/jcla.23756 33734490 PMC8128308

[20] Shademan B Nourazarian A Laghousi D Karamad V Nikanfar M. Exploring potential serum levels of Homocysteine, interleukin-1 beta, and apolipoprotein B 48 as new biomarkers for patients with ischemic stroke. J Clin Lab Anal. (2021) 35:e23996. 10.1002/jcla.23996 34492129 PMC8551691

[21] Yurekli UF Tunc Z. Correlation between Vitamin D, homocysteine and brain-derived neurotrophic factor levels in patients with ischemic stroke. Eur Rev Med Pharmacol Sci. (2022) 26:8004–10. 36394751 10.26355/eurrev_202211_30154

[22] Wang J Gu J Huang Y Fang Y Lin J. The association between serine hydroxymethyl transferase 1 gene hypermethylation and ischemic stroke. Bosn J Basic Med Sci. (2021) 21(4):454-460. 10.17305/bjbms.2020.5188 33259775 PMC8292870

[23] Kawamoto R Kajiwara T Oka Y Takagi Y. An association between plasma homocysteine concentrations and ischemic stroke in elderly Japanese. J Atheroscler Thromb. (2002) 9:121–5. 10.5551/jat.9.121 12236316

[24] Yoldas T Gonen M Godekmerdan A Ilhan F Bayram E. The serum high-sensitive C reactive protein and homocysteine levels to evaluate the prognosis of acute ischemic stroke. Mediators Inflamm. (2007) 2007:15929. 10.1155/2007/15929 17597836 PMC1892643

[25] Salem-Berrabah OB Mrissa R Machghoul S Hamida AB N'siri B Mazigh C . Hyperhomocysteinemia, C677T MTHFR polymorphism and ischemic stroke in Tunisian patients. Tunis Med. (2010) 88:655–9. 20812180

[26] Omrani HQ Shandiz EE Qabai M Chaman R Fard HA Qaffarpoor M. Hyperhomocysteinemia, folateo, and B12 vitamin in Iranian patients with acute ischemic stroke. ARYA Atheroscler. (2011) 7:97–101. 22577454 PMC3347852

[27] Wei GJ Yuan MQ Jiang LH Lu YL Liu CH Luo HC . A genetic variant of miR-34a contributes to susceptibility of ischemic stroke among Chinese population. Front Physiol. (2019) 10:432. 10.3389/fphys.2019.00432 31068831 PMC6491571

[28] Luo HC Luo QS Wang CF Lei M Li BL Wei YS. Association of miR-146a, miR-149, miR-196a2, miR-499 gene polymorphisms with ischemic stroke in a Chinese people. Oncotarget. (2017) 8:81295–304. 10.18632/oncotarget.18333 29113388 PMC5655283

[29] Modi M Prabhakar S Majumdar S Khullar M Lal V Das CP. Hyperhomocysteinemia as a risk factor for ischemic stroke: an Indian scenario. Neurol India. (2005) 53:297–302. 10.4103/0028-3886.16927 16230796

[30] Xiao J Yuan Q Zhang S Li X Bai H Wang Y . The telomere length of peripheral blood cells is associated with the risk of ischemic stroke in Han population of northern China. Medicine. (2019) 98:e14593. 10.1097/MD.0000000000014593 30762812 PMC6408041

[31] Narayan S Chandrasekaran A Basu D Hanumanthappa N Aghoram R Dutta TK Rejul V. Prothrombotic factors have significant association with arterial and venous strokes in Indian Tamilians. J Appl Lab Med. (2021) 6:101–12. 10.1093/jalm/jfaa198 33313850

[32] Al-Allawi NA Avo AS Jubrael JM. Methylenetetrahydrofolate reductase C677T polymorphism in Iraqi patients with ischemic stroke. Neurol India. (2009) 57:631–5. 10.4103/0028-3886.57821 19934565

[33] Lu SJ Zhou XS Zheng Q Chen HL Geng YL. Platelet membrane receptor P2Y12 H1/H2 polymorphism is highly associated with cerebral infarction: a case-control study. Neuropsychiatr Dis Treat. (2018) 14:2225–31. 10.2147/NDT.S171213 30214212 PMC6121754

[34] Zheng LJ Lin X Xue YJ. Effect of cerebral ischemic strokes in different cerebral artery regions on left ventricular function. Front Cardiovasc Med. (2022) 9:782173. 10.3389/fcvm.2022.782173 35345487 PMC8957275

[35] Chen C Qiao X Guo J Yang T Wang M Ma Y . Related factors based on non-targeted metabolomics methods in minor ischaemic stroke. Front Endocrinol (Lausanne). (2022) 13:952918. 10.3389/fendo.2022.952918 36237188 PMC9552842

[36] Zhou X Qi L. miR-124 is downregulated in serum of acute cerebral infarct patients and shows diagnostic and prognostic value. Clin Appl Thromb Hemost. (2021) 27:10760296211035446. 10.1177/10760296211035446 34702084 PMC8554555

[37] Chen QY Liu N Ma J Fang Y Cao Y Li H . Effect of a pre-microRNA-149 (miR-149) genetic variation on the risk of ischemic stroke in a Chinese Han population. Genet Mol Res. (2015) 14:2582–9. 10.4238/2015.March.30.17 25867405

[38] Veeranki S Gandhapudi SK Tyagi SC. Interactions of hyperhomocysteinemia and T cell immunity in causation of hypertension. Can J Physiol Pharmacol. (2017) 95:239–46. 10.1139/cjpp-2015-0568 27398734 PMC5519337

[39] Herrmann W Obeid R. Homocysteine: a biomarker in neurodegenerative diseases. Clin Chem Lab Med. (2011 M) 49:435–41. 10.1515/CCLM.2011.084 21388339

[40] Morris AA KoŽich V Santra S Andria G Ben-Omran TI Chakrapani AB . Guidelines for the diagnosis and management of cystathionine beta-synthase deficiency. J Inherit Metab Dis. (2017) 40:49–74. 10.1007/s10545-016-9979-0 27778219 PMC5203861

[41] Harris S Rasyid A Kurniawan M Mesiano T Hidayat R. Association of high blood homocysteine and risk of increased severity of ischemic stroke events. Int J Angiol. (2019) 28:34–8. 10.1055/s-0038-1667141 30880891 PMC6417904

[42] Hankey GJ Eikelboom JW. Homocysteine and stroke. Curr Opin Neurol. (2001) 14:95–102. 10.1097/00019052-200102000-00015 11176224

[43] Boldyrev A Bryushkova E Mashkina A Vladychenskaya E. Why is homocysteine toxic for the nervous and immune systems? Curr Aging Sci. (2013) 6:29–36. 10.2174/18746098112059990007 23237596

[44] Ziemińska E Stafiej A Łazarewicz JW. Role of group I metabotropic glutamate receptors and NMDA receptors in homocysteine-evoked acute neurodegeneration of cultured cerebellar granule neurones. Neurochem Int. (2003) 43:481–92. 10.1016/S0197-0186(03)00038-X 12742095

[45] Rabelo NN Telles JPM Pipek LZ Farias Vidigal Nascimento R Gusmão RC Teixeira MJ . Homocysteine is associated with higher risks of ischemic stroke: a systematic review and meta-analysis. PLoS ONE. (2022) 17:e0276087. 10.1371/journal.pone.0276087 36227950 PMC9560514

[46] Zhang T Jiang Y Zhang S Tie T Cheng Y Su X . The association between homocysteine and ischemic stroke subtypes in Chinese: a meta-analysis. Medicine. (2020) 99:e19467. 10.1097/MD.0000000000019467 32195946 PMC7220264

[47] Yu B Yang P Xu X Shao L. C-reactive protein for predicting all-cause mortality in patients with acute ischemic stroke: a meta-analysis. Biosci Rep. (2019) 39:BSR20181135. 10.1042/BSR20181135 30718369 PMC6379508

[48] Ikram MA Wieberdink RG Koudstaal PJ. International epidemiology of intracerebral hemorrhage. Curr Atheroscler Rep. (2012) 14:300–6. 10.1007/s11883-012-0252-1 22538431 PMC3388250

[49] Zhou Z Liang Y Qu H Zhao M Guo F Zhao C Teng W. Plasma homocysteine concentrations and risk of intracerebral hemorrhage: a systematic review and meta-analysis. Sci Rep. (2018) 8:2568. 10.1038/s41598-018-21019-3 29416106 PMC5803270

[50] Sato S Uehara T Hayakawa M Nagatsuka K Minematsu K Toyoda K. Intra- and extracranial atherosclerotic disease in acute spontaneous intracerebral hemorrhage. J Neurol Sci. (2013) 332:116–20. 10.1016/j.jns.2013.06.031 23859180

[51] Boysen G Brander T Christensen H Gideon R Truelsen T. Homocysteine and risk of recurrent stroke. Stroke. (2003) 34:1258–61. 10.1161/01.STR.0000069017.78624.37 12702838

[52] Del Ser T Barba R Herranz AS Seijas V López-Manglano C Domingo J . Hyperhomocyst(e)inemia is a risk factor of secondary vascular events in stroke patients. Cerebrovasc Dis. (2001) 12:1–98. 10.1159/000047687 11490102

[53] Jakubowski H. Pathophysiological consequences of homocysteine excess. J Nutr. (2006) 136:1741S−9S. 10.1093/jn/136.6.1741S 16702349

[54] VITATOPS Trial Study Group. B vitamins in patients with recent transient ischaemic attack or stroke in the VITAmins TO Prevent Stroke (VITATOPS) trial: a randomized, double-blind, parallel, placebo-controlled trial. Lancet Neurol. (2010) 9:855–65. 10.1016/S1474-4422(10)70187-3 20688574

[55] Hankey GJ Eikelboom JW Yi Q Lees KR Chen C Xavier D . VITATOPS trial study group. Antiplatelet therapy and the effects of B vitamins in patients with previous stroke or transient ischaemic attack: a post-hoc subanalysis of VITATOPS, a randomized, placebo-controlled trial. Lancet Neurol. (2012) 11:512–20. 10.1016/S1474-4422(12)70091-1 22554931 PMC3361667

[56] Toole JF Malinow MR Chambless LE Spence JD Pettigrew LC Howard VJ . Lowering homocysteine in patients with ischemic stroke to prevent recurrent stroke, myocardial infarction, and death: the Vitamin Intervention for Stroke Prevention (VISP) randomized controlled trial. JAMA. (2004) 291:565–75. 10.1001/jama.291.5.565 14762035

[57] Spence JD. Homocysteine: call off the funeral. Stroke. (2006) 37:282–3. 10.1161/01.STR.0000199621.28234.e2 16397178

[58] Rajan S Wallace JI Brodkin KI Beresford SA Allen RH Stabler SP. Response of elevated methylmalonic acid to three dose levels of oral cobalamin in older adults. J Am Geriatr Soc. (2002) 50:1789–95. 10.1046/j.1532-5415.2002.50506.x 12410896

[59] Yuan M Wang B Tan S. Mecobalamin and early functional outcomes of ischemic stroke patients with H-type hypertension. Rev Assoc Med Bras. (1992). (2018) 64:428–32. 10.1590/1806-9282.64.05.428 30304141

[60] Zeng R Xu CH Xu YN Wang YL Wang M. The effect of folate fortification on folic acid-based homocysteine-lowering intervention and stroke risk: a meta-analysis. Public Health Nutr. (2015) 18:1514–21. 10.1017/S1368980014002134 25323814 PMC10271370

[61] Huang T Chen Y Yang B Yang J Wahlqvist ML Li D. Meta-analysis of B vitamin supplementation on plasma homocysteine, cardiovascular and all-cause mortality. Clin Nutr. (2012) 31:448–54. 10.1016/j.clnu.2011.01.003 22652362

