# Corrigendum: The role of homocysteine levels as a risk factor of ischemic stroke events: a systematic review and meta-analysis

**DOI:** 10.3389/fneur.2024.1404808

**Published:** 2024-04-04

**Authors:** Rizaldy Taslim Pinzon, Vincent Ongko Wijaya, Vanessa Veronica

**Affiliations:** ^1^Faculty of Medicine, Duta Wacana Christian University, Yogyakarta, Indonesia; ^2^Neurology Department, Bethesda Hospital, Yogyakarta, Indonesia

**Keywords:** homocysteine, ischemic stroke, risk factor, systematic review, meta-analysis

In the published article, there was an error in the usage of abbreviation in a measurement unit.

A correction has been made to the Discussion section, paragraph 6. This sentence previously stated:

“A dose–response study revealed that geriatric patients with cobalamin levels below 221 pmol/L require 1000 g daily for optimal absorption (58).”

The corrected sentence appears below:

“A dose–response study revealed that geriatric patients with cobalamin levels below 221 pmol/L require 1000 mcg daily for optimal absorption (58).”

The authors apologize for this error and state that this does not change the scientific conclusions of the article in any way. The original article has been updated.

